# Serum from Stroke Patients with High-Grade Carotid Stenosis Promotes Cyclooxygenase-Dependent Endothelial Dysfunction in Non-ischemic Mice Carotid Arteries

**DOI:** 10.1007/s12975-022-01117-1

**Published:** 2022-12-19

**Authors:** Lídia Puertas-Umbert, Núria Puig, Mercedes Camacho, Ana Paula Dantas, Rebeca Marín, Joan Martí-Fàbregas, Elena Jiménez-Xarrié, Sonia Benitez, Pol Camps-Renom, Francesc Jiménez-Altayó

**Affiliations:** 1 Department of Pharmacology, Therapeutics and Toxicology, School of Medicine, Universitat Autònoma de Barcelona, Barcelona, Spain; 2 Neuroscience Institute, Universitat Autònoma de Barcelona, Barcelona, Spain; 3 Institut d’Investigació Biomèdica Sant Pau (IIB, SANT PAU), Barcelona, Spain; 4 CIBER de Enfermedades Cardiovasculares (CIBERCV), Madrid, Spain; 5 Department of Molecular Biology and Biochemistry, School of Medicine, Universitat Autònoma de Barcelona, Barcelona, Spain; 6 Institut d’Investigacions Biomèdiques August Pi I Sunyer (IDIBAPS), Cardiovascular Institute, Hospital Clinic, University of Barcelona, Barcelona, Spain; 7 Department of Neurology, IIB SANT PAU, Hospital de La Santa Creu i Sant Pau, Barcelona, Spain; 8 CIBER of Diabetes and Related Metabolic Diseases (CIBERDEM), Instituto de Salud Carlos III, Madrid, Spain

**Keywords:** Atherosclerosis, Carotid plaque, Ischemic stroke, High-grade carotid artery stenosis, Endothelial dysfunction, Inflammatory biomarkers

## Abstract

Atherosclerosis is responsible for 20% of ischemic strokes, and severe carotid stenosis is associated with a higher incidence of first-ever and recurrent strokes. The release of pro-inflammatory mediators into the blood in severe atherosclerosis may aggravate endothelial dysfunction after stroke contributing to impair disease outcomes. We hypothesize that environments of severe carotid atherosclerotic disease worsen endothelial dysfunction in stroke linked to enhanced risk of further cerebrovascular events. We mounted nonischemic common carotid arteries from 2- to 4-month-old male Oncins France 1 mice in tissue baths for isometric contraction force measurements and exposed them to serum from men with a recent ischemic stroke and different degrees of carotid stenosis: low- or moderate-grade stenosis (LMGS; < 70%) and high-grade stenosis (HGS; ≥ 70%). The results show that serum from stroke patients induced an impairment of acetylcholine relaxations in mice carotid arteries indicative of endothelium dysfunction. This effect was more pronounced after incubation with serum from patients with a recurrent stroke or vascular death within 1 year of follow-up. When patients were stratified according to the degree of stenosis, serum from HGS patients induced more pronounced carotid artery endothelial dysfunction, an effect that was associated with enhanced circulating levels of IL-1β. Mechanistically, endothelial dysfunction was prevented by both nonselective and selective COX blockade. Altogether, the present findings add knowledge on the understanding of the mechanisms involved in the increased risk of stroke in atherosclerosis and suggest that targeting COX in the carotid artery wall may represent a potential novel therapeutic strategy for secondary stroke prevention.

**Supplementary Information:**

The online version contains supplementary material available at 10.1007/s12975-022-01117-1.

## Introduction

Ischemic stroke is the second leading cause of mortality and a major cause of morbidity worldwide. Current trends indicate that its burden will further increase, due to an aging population and a high prevalence of attributable risk factors [1]. Despite advances in secondary prevention therapies, the risk of recurrence still remains high [2]. Atherosclerosis is an important risk factor for ischemic stroke and is associated with a higher risk of recurrence compared to other stroke subtypes [3]. Indeed, atherosclerotic plaques, mainly in the internal carotid artery, are responsible for an estimate of 20% of acute ischemic stroke episodes [4]. Current evidence indicates that plaque morphological features, intraplaque neovascularization, and plaque inflammation are important predictors of stroke, though the degree of carotid plaque stenosis remains a key biomarker for the development of cerebrovascular events [5, 6]. For instance, around 10–15% of all strokes follow thromboembolism from a prior asymptomatic internal carotid artery stenosis > 50% [7]. In addition, patients with severe carotid stenosis ≥ 70% face a twofold increased risk of stroke vs. moderate stenosis [8] and a threefold increased risk of early recurrent stroke if plaque is not removed [9]. Furthermore, the degree of carotid stenosis is the main factor influencing decision-making to undergo pharmacological vs. surgical management of the plaque [5]. Nonetheless, the factors and mechanisms involved in the augmented risk of poorer poststroke outcomes in patients with severe carotid narrowing are not fully understood.

Atherosclerotic plaques develop after a multifaceted sequence of events that include endothelial dysfunction, lipoprotein entry leading to lipid deposition into the arterial wall, smooth muscle cell proliferation, and excessive extracellular matrix deposition. In this scenario, an abnormal lipid profile, especially the abundance of modified forms of low-density lipoproteins, is a risk factor for atherosclerosis, owing to its role in inflammation, intracellular lipid accumulation, and vascular function [10]. Likewise, inflammation plays a critical role in both the initiation and progression of the atherosclerotic plaque [11, 12]. With time, larger plaques can narrow the carotid artery lumen and, in inflammatory environments, can become vulnerable/unstable and prone to fissure, erosion, or rupture resulting in thromboembolic events [11]. Moreover, inflammatory mediators within the plaque can be released into the circulation to propagate inflammation [13]. Besides, previous studies showed that fluctuations in serum concentrations of certain cytokines, chemokines, and growth factors released after stroke were positively correlated with poorer disease outcomes in acute ischemic stroke patients [14]. In this regard, IL-1β plays a key role by promoting inflammation and impaired vessel function. Accordingly, the involvement of this cytokine in atherosclerosis has been demonstrated in the large clinical trial CANTOS [15, 16]. In addition, several pro-inflammatory cytokines, chemokines, adhesion molecules, and metalloproteases are significantly elevated in the plasma of patients with a recent ischemic stroke and carotid atherosclerosis, and some of them [fractalkine (FKN), soluble vascular cell adhesion molecule-1 (sVCAM-1), and soluble intercellular adhesion molecule-1 (sICAM-1)] are independently associated with the degree of plaque inflammation [17]. Therefore, in an environment of carotid atherosclerotic disease where inflammation is already present, stroke-induced release of pro-inflammatory factors may enhance plaque inflammation leading to an increased risk of plaque rupture.

Pro-inflammatory molecules are known to cause endothelial dysfunction [18], which, in turn, is an early marker of atherosclerosis [12, 13]. The endothelium is a chief contributor to vascular homeostasis and is responsible for the synthesis of multiple factors that modulate vascular smooth muscle tone and, consequently, blood flow. Under physiological conditions, the endothelium is balanced in favor of vasodilation, anticoagulation, and anti-inflammatory profile. However, in some pathological conditions with a well-recognized inflammatory component, the release of relaxing and contracting factors is either reduced or increased, respectively, resulting in endothelial dysfunction [12]. Previous studies showed that exposure of arteries from healthy non-ischemic rats with serum from ischemic stroke patients can promote endothelial dysfunction [19, 20]. This translational approach provides an interesting platform for the study of the vasoactive effects of circulating molecules released after stroke, as it may contribute to a better understanding of the underlying mechanisms involved in the development of poorer clinical outcomes. Nonetheless, the influence of carotid stenosis grading on the release of circulating inflammatory molecules and their potential effects on poststroke carotid artery reactivity has not been elucidated.

Atherosclerosis is linked to abnormal cyclooxygenase (COX)-derived prostanoids production both in humans and mice [21]. COX is present in two isoforms, COX-1, which is the constitutive enzyme, but its levels can be upregulated in certain conditions, and COX-2, which is generally induced by different stimuli such as pro-inflammatory cytokine exposure, though it is also constitutively expressed in some tissues, including the brain [21]. Prostacyclin and thromboxane A2 are two major COX-derived prostanoids with antagonistic effects that are essential mediators in the cardiovascular system, and their imbalance has been linked to endothelial dysfunction leading to cardiovascular disease [21]. Prostacyclin is a potent inhibitor of platelet activation and a vasodilatory mediator that in healthy vessels is mainly produced by endothelial COX-1 [21]. This pathway is generally associated with protection, though a growing body of evidence suggest that COX-1 is also a pathological driver of neuroinflammatory [22] and cardiovascular [23] disease. Alternatively, thromboxane A2 is a pro-thrombotic, proinflammatory, and vasoconstrictive mediator that in physiological conditions is primarily produced by COX-1 located in platelets, macrophages, and endothelial cells, whereas during inflammation it is thought to derive mainly from COX-2 [21].

In the present study, we hypothesize that in an environment of severe carotid atherosclerotic disease, pro-inflammatory factors released from the vulnerable plaque worsen endothelial dysfunction after stroke through a COX-dependent pathway, a detrimental effect that increases the risk of further cerebrovascular events. To this aim, we exposed common carotid arteries from nonischemic male mice to serum from men with a recent ischemic stroke and varying degrees of carotid stenosis.

## Materials and Methods

### Patients

Men with an anterior circulation ischemic stroke or transient ischemic attack and carotid atherosclerosis (*n* = 30) and sex- and age-matched healthy (control) subjects (*n* = 20) were recruited between January 2016 and March 2019 to participate in the observational cohort study (NCT03218527) at Hospital de la Santa Creu i Sant Pau (Barcelona, Spain). Low recruitment of women (25%) in our cohort did not allow their inclusion in the study. The study was approved by the hospital’s ethics committee (approval code: IIBSP-LRB-2017–54), the patients gave written consent to participate, and human serum studies were performed in accordance with the Helsinki Declaration of 1975 and the *Ley de Investigación Biomédica* 14/2007 from the Spanish Ministry.

Patients were enrolled in the study if they fulfilled the following criteria: (i) age ≥ 50 years; (ii) anterior circulation ischemic stroke or transient ischemic attack within 7 days before inclusion; (iii) at least one atherosclerotic plaque in the internal carotid artery on the side consistent with stroke symptoms, regardless of the degree of stenosis; and (iv) poststroke modified Rankin Scale score < 4. Patients were excluded if (i) they showed a definitive cardioembolic cause of stroke or an unusual etiology according to the trial of ORG 10,172 in acute stroke treatment criteria [24]; (ii) had prior neck irradiation or ipsilateral carotid surgery/stenting; or (iii) presented concomitant systemic infection at the time of blood extraction.

The following variables were collected for all of the patients at admission: (i) age and sex; (ii) past medical history including hypertension, diabetes, dyslipidemia, prior stroke/transient ischemic attack, and coronary artery disease; (iii) tobacco and alcohol consumption; (iv) previous treatments; (v) poststroke modified Rankin Scale score and National Institute of Health Stroke Scale score; (vi) stroke etiology after the diagnostic work-up including at least a 24-h electrocardiogram, echocardiogram, and ultrasound carotid examination; and (viii) results of blood test including lipid profile (Table 1). In addition, all patients had a brain imaging with computed tomography and/or magnetic resonance. Carotid stenosis was graded using the North American Symptomatic Carotid Endarterectomy Trial approach in patients with a computed tomography-angiography available and by hemodynamic criteria using ultrasound.

**Table 1:** Clinical characteristics of patients

*Baseline characteristics*	Control (*n* = 20)	Stroke patients (total) (*n* = 30)	Stroke patients		*P* value
			LMGS (*n* = 20)	HGS (*n* = 10)	
Age (mean ± SEM)	73.8 ± 1.3	73.9 ± 2.0	72.0 ± 2.3	77.7 ± 3.6	0.174
Current smoking, % (*n*)	0 (0)	77 (23)	80 (16)	80 (8)	1
Hypertension, % (*n*)	75 (15)	83 (25)	95 (19)	60 (6)^*^	0.015
Diabetes, % (*n*)	20 (4)	50 (15)	45 (9)	60 (6)	0.439
Obesity, % (*n*)	10 (2)	13 (4)	15 (3)	5 (1)	0.704
Dyslipidemia, % (*n*)	25 (5)	60 (18)	55 (11)	70 (7)	0.429
Alcohol, % (*n*) Carotid revascularization, % (*n*)	0 (0) n/a	13 (4) 16.7 (5)	15 (3) 0 (0)	5 (1) 50 (5)^*^	0.704 0.018
NIHSS score at admission, median [IQR]	n/a	2.5 [1.00–4.75]	3.0 [1.00 $-5.00$]	2.0 [0 $-$ 4.0]	0.409
Glucose [mg/dl] (mean ± SEM)	-	137.1 ± 11.0	144.3 ± 14.4	122.7 ± 16.2	0.486
Total cholesterol [mg/dl] (mean ± SEM)	181.6 ± 7.9	147.4 ± 6.7	147.8 ± 9.7	146.7 ± 7.4	0.939
Triglycerides [mg/dl] (mean ± SEM)	123.5 ± 13.65	108.5 ± 7.4	98.6 ± 9.6	125.4 ± 10.0 ^**#**^	0.021
HDL cholesterol [mg/dl] (mean ± SEM)	50.32 ± 2.3	38.5 ± 2.2	40.0 ± 2.9	36.3 ± 2.4	0.383
LDL cholesterol [mg/dl] (mean ± SEM)	104.9 ± 7.9	88.6 ± 6.1	90.7 ± 9.2	85.1 ± 6.7	0.667
*Follow-up characteristics*					
Recurrent stroke or vascular death, % (*n*)	n/a	37 (11)	25 (5)	60 (6)	0.108

*n/a*, not applicable; NIHSS, National institute of Health stroke scale; HDL, high-density lipoprotein; LDL, low-density lipoprotein.^*^P < 0.05 *vs. *LMGS by *χ*^*2*^test; ^# ^*P* < 0.05 *vs. *LMGS by unpaired Student’s *t *test

Healthy controls were recruited during the same period, were older than 50 years, and presented no prior cardiovascular disease. A carotid ultrasound was performed routinely to all healthy subjects to rule out the presence of asymptomatic high degree stenosis in both the internal carotid artery and an electrocardiogram to rule out silent ischemic heart disease and atrial fibrillation.

### Outcome and Follow-Up

Standardized follow-up assessments were performed on day 90 and, from then, every 6 months until the study terminated (1-year follow-up). During the follow-up, we registered any recurrent ischemic stroke defined as a new sudden onset of persistent or transient neurological deficit or death due to suspected vascular origin. Recurrent strokes were assessed by a stroke neurologist to minimize the inclusion of stroke mimics.

### Serum and Plasma Collection

After giving informed consent, blood samples were collected by venipuncture within 7 days (*n* = 30) and at 1 year of follow-up (long-term; *n* = 6) after ischemic stroke onset. Samples were centrifuged at 2000 × *g* (4ºC; 15 min), aliquoted, and stored at − 80ºC until analyzed. Serum and plasma samples were divided into two groups characterized by the degree of internal carotid artery stenosis (measured at admission by Doppler ultrasound or computed tomography angiography): (i) low- or moderate-grade stenosis (LMGS; < 70%) and (ii) high-grade stenosis (HGS; ≥ 70%). Also, an extra blood tube was extracted and sent to Sant Pau Central Laboratory Analysis for routine biochemical determinations (Table 1).

### Animals

Male non-consanguineous Oncins France 1 mice (2 to 4-months-old, *n* = 62) were obtained from Charles River (Sant Cugat del Vallès, Spain). Mice were housed at the animals’ facility under constant temperature (20 ± 2ºC) and humidity conditions, 12:12-h dark/light cycle, and provided with food and water ad libitum. All procedures were performed in accordance with the Spanish Legislation (RD 53/2013) and with the Guide for the Care and Use of Laboratory Animals, published by the US National Institutes of Health (NIH Publication 85–23, revised 1996). Experiments were approved by the Ethics Committee of the Universitat Autònoma de Barcelona (approval code: FJA-eut/01).

### Tissue Preparation

Mice were euthanized under isoflurane (5% mixed with 0.8 L/min O_2_) by exsanguination via direct cardiac puncture. Common carotid arteries were gently dissected and placed in oxygenated (95% O_2_, 5% CO_2_) icecold Krebs–Henseleit solution (KHS; NaCl 112 mM, KCl 4.7 mM, CaCl_2_ 2.5 mM, KH_2_PO_4_ 1.2 mM, MgSO_4_ 1.2 mM, NaHCO_3_ 25 mM and glucose 11.1 mM) and cleaned free of fat and connective tissue.

### Carotid Artery Reactivity

Carotid segments (1.5 mm in length) were mounted onto a four-channel wire (40 µm) myograph (model 620 M; Danish Myo Technology, Aarhus, Denmark) under isometric conditions, filled with ice-cold KHS and gassed with 95% O_2_ and 5% CO_2_, as reported [25]. After a 30-min stabilization period at 37ºC, arteries were stretched to a resting tension of 2.45 mN, as described [26]. Then, arteries were allowed to equilibrate for a further 45 min and were exposed twice to a K^+^-enriched KHS (containing 100 mM KCl). After washing, vessels were preincubated (45 min) in the absence (no serum; internal control) or presence (1%, 3%, and 10% volume/volume in KHS) of serum from control healthy donors or ischemic stroke patients. Endothelial-dependent vasodilatations to acetylcholine (ACh; 0.1 nM-10 µM) were assessed in arteries precontracted with the thromboxane A_2_ stable analog 9,11-dideoxy-11α,9αepoxymethanoprostaglandin F2α (U46619) to achieve 70–100% of KCl 100-mM contraction. Preliminary studies demonstrated that right and left common carotid arteries were equally responsive to ACh (results not shown).

To remove the influence of nitric oxide synthase (NOS) and cyclooxygenase (COX), the effects of the nonselective NOS inhibitor Nω-nitro-l-arginine methyl ester (L-NAME; 300 µM), the nonselective COX inhibitor indomethacin (10 µM), or COX-1 and COX-2 selective inhibitors SC-560 (0.3 µM) [27] and NS-398 (1 µM) [28], respectively, were determined by adding each treatment 30 min before ACh-induced responses. It is described that more than 95% of COX-1 activity is inhibited with 0.3 µM SC-560, whereas more than 40% of COX-2 activity remains up to 100 µM SC-560 [27]. Besides, the NS-398 inhibitory concentration 50 values for COX-1 are > 100 μM [28]. Finally, to study smooth-muscle sensitivity to NO, arteries were exposed to increasing concentrations of the NO donor sodium nitroprusside (SNP; 0.1–100 µM).

### Measurements of Circulating Inflammatory Molecule Concentrations in Plasma

Plasma concentrations of sICAM-1, sVCAM-1, FKN, interleukin (IL)-1 β, IL-6, and tumor necrosis factor (TNF)-α, which have been previously involved in endothelial dysfunction, were measured using Luminex xMAP® technology with a MILLIPLEX® MAP multiplexed assay kit (Merck Millipore, Burlington, Massachusetts, USA) following the protocol provided by the manufacturer, as described [17]. The xPONENT version 3.1 (Luminexcorp) and MILLIPLEX® Analyst Version 3.5 (Merck Millipore) were used for acquisition and data analysis, respectively.

### Statistical Analyses

Characteristics of the patients are presented as mean ± standard error of the mean (SEM) for continuous variables, as median [interquartile range (IQR)] for skewed continuous variables and as percentages for categorical variables. Patients were divided according to the degree of carotid stenosis into two groups: HGS ≥ 70% and LMGS 30 and 69%. Comparisons between patients and healthy subjects and between patients with HGS vs. LMGS were performed using the Student’s *t* test or Kruskal–Wallis test for continuous variables and the Chi-square test for categorical variables.

Animals’ data are expressed as mean ± SEM of the number (*n*) of animals in each group (shown in figure legends). Vasodilator responses to ACh and SNP are expressed as a percentage of the tone generated by U46619 pre-contractions. Nonlinear regression (sigmoid curve fitting, variable slope) was performed to obtain maximal responses (*Emax*) and sensitivity (*pEC50*) in the vasodilator responses curves to ACh and SNP. Testing for normality was systematically performed before selecting the appropriate statistical test. Student’s *t* test was used to compare two groups and one-way ANOVA to compare three or more groups. Statistical analysis was performed with GraphPad Prism version 8.3 (San Diego, California, USA) software. Statistical significance was set as *P* < 0.05.

## Results

### Serum from Stroke Patients Impairs ACh-Induced Relaxations, Which Is More Pronounced After Incubation with Serum from Patients with a Vascular Event

**Fig. 1:**
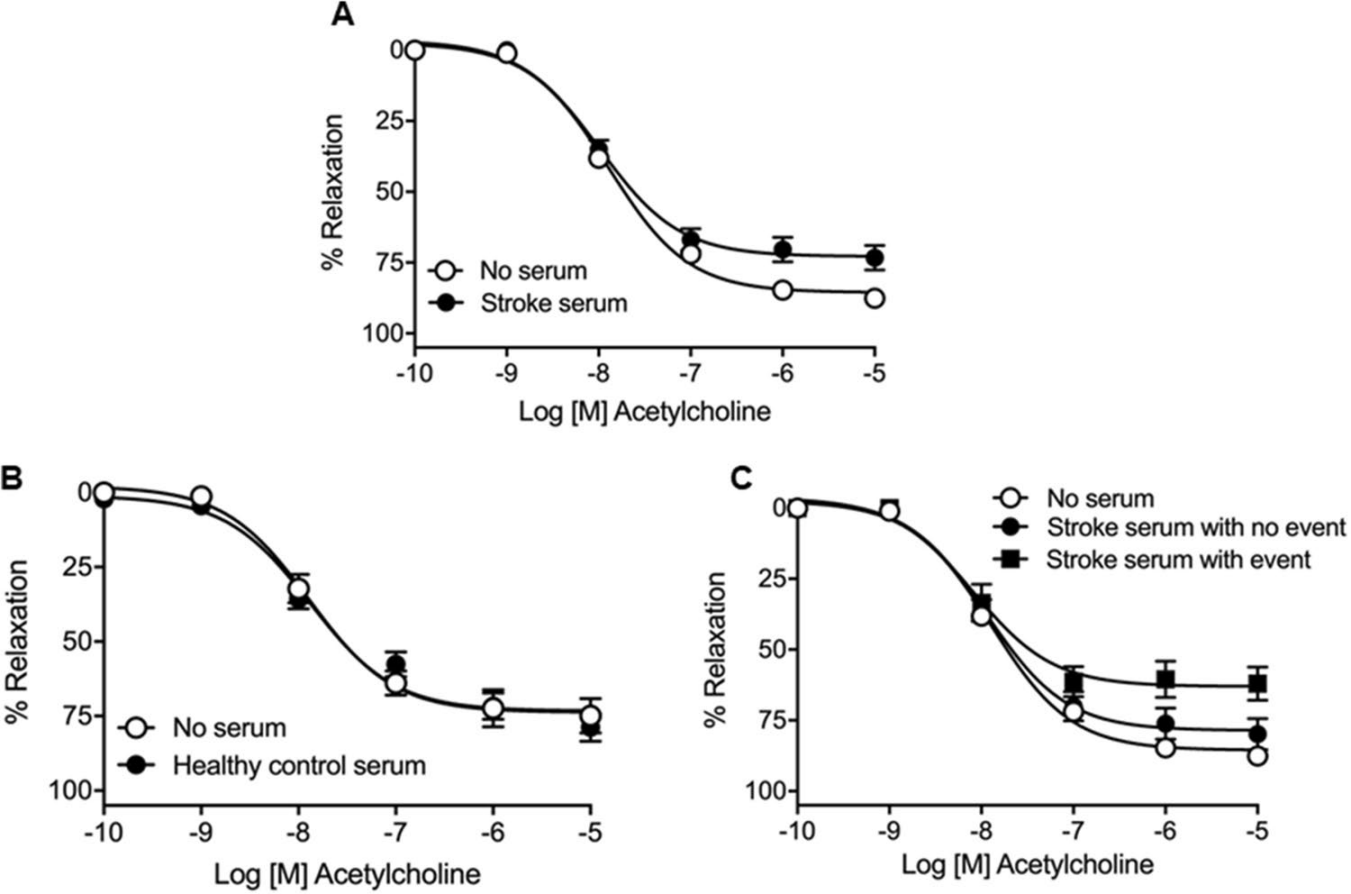
Effects of ischemic stroke serum on acetylcholine relaxations in male OF-1 mice common carotid arteries. Concentration–response curves to acetylcholine in mice carotid arteries incubated in the absence (no serum) or presence (10%) of ischemic stroke serum (**A**), healthy control serum (**B**), or serum from stroke patients that suffered or not from an event (stroke recurrence or vascular death) (**C**). The results are mean ± SEM for *n* = 11–26 (no serum), *n* = 20 (healthy control serum), *n* = 30 (acute stroke serum), *n* = 19 (no event), *n* = 11 (event)

Clinical features of stroke patients and age- and sex-matched healthy controls are shown in Table 1. Endothelium-dependent ACh-induced relaxations were significantly reduced (i.e., lower *Emax*; *P* < 0.0001) in mice carotid arteries incubated with 10% serum from patients with a recent (within 7 days from stroke onset) ischemic stroke, compared to arteries that were not exposed to serum (Fig. 1A; Table 2) or arteries that were exposed to 1% and 3% stroke serum (Supplementary Fig. 1; Supplementary Table 1). Consequently, concentration of 10% of serum was chosen for further experiments. To determine whether this effect was associated with a xenogeneic response to human serum, we incubated mice carotid arteries with serum from healthy donor patients, which did not cause any effect on ACh responses as compared with arteries not exposed to serum (Fig. 1B; Supplementary Table 2). Therefore, we used arteries not incubated with serum as internal control group for further experiments.

**Table 2:** Potency (*pEC*_*50*_) and maximal response (*E*_*max*_) were obtained from concentration–response curves of acetylcholine (ACh) in mice carotid arteries in the absence (no serum) or presence (10%) of stroke serum

	No serum (26)	Stroke serum (30)
*pEC*_*50*_	7.89 ± 0.05	7.99 ± 0.09
*E*_*max*_	85.54 ± 1.49	72.81 ± 2.04****

The results are mean ± SEM and number of vessels is shown in parentheses. ^****^P < 0.0001 versus no serum by unpaired Student’s *t* test

To explore the potential translational significance of the stroke serum-induced impairment of ACh responses in mice carotid arteries, samples were divided in two cohorts based on patient’s outcome, i.e., occurrence or not of vascular event (stroke recurrence or vascular death) during follow-up. The results show that serum from stroke patients with a vascular event induced greater ACh vasodilator dysfunction (i.e., lower *Emax*) than serum from stroke patients with no vascular events during the follow-up (*P* < 0.0001 stroke serum with event vs. no serum; *P* < 0.001 stroke serum with event vs. stroke serum with no event) (Fig. 1C; Table 3).

**Table 3:** Potency (*pEC*_*50*_) and maximal response (*E*_*max*_) were obtained from concentration–response curves of acetylcholine (ACh) in mice carotid arteries in the absence (no serum) or presence (10%) of serums from patients who suffered or not from an event (stroke recurrence or vascular death)

	No serum (26)	Stroke serum with no event (19)	Stroke serum with event (11)
*pEC*_*50*_	7.89 ± 0.05	7.94 ± 0.10	8.09 ± 0.15
*E*_*max*_	85.54 ± 1.49	78.56 ± 2.58^*^	63.03 ± 3.04^****###^

The results are mean ± SEM and number of vessels is shown in parentheses. ^*^P < 0.05; ^****^P < 0.0001 *vs.* no serum; ^###^*P* < 0.001 *vs.* stroke serum with no event by 1-way ANOVA followed by Tukey’s posttest

### Serum from HGS Stroke Patients Induces a Greater Impairment of Carotid Artery Vasodilation Than Serum from LMGS Stroke Patients

To investigate the influence of carotid plaque size on ACh vasodilator dysfunction induced by stroke serum, samples were divided in two groups based on the following grade of carotid artery stenosis: < 70% stenosis (LMGS serum) and ≥ 70% stenosis (HGS serum). Hypertension was more frequent in the LMGS *vs.* HGS group (Table 1). In addition, levels of triglycerides were significantly higher in HGS *vs.* LMGS stroke patients. Of note, compared to the LMGS group, HGS stroke patients showed a higher percentage (60% *vs.* 25%) of incidence of recurrent stroke or vascular death and a significantly higher percentage (50% *vs.* 0%) of carotid revascularization.

**Fig. 2:**
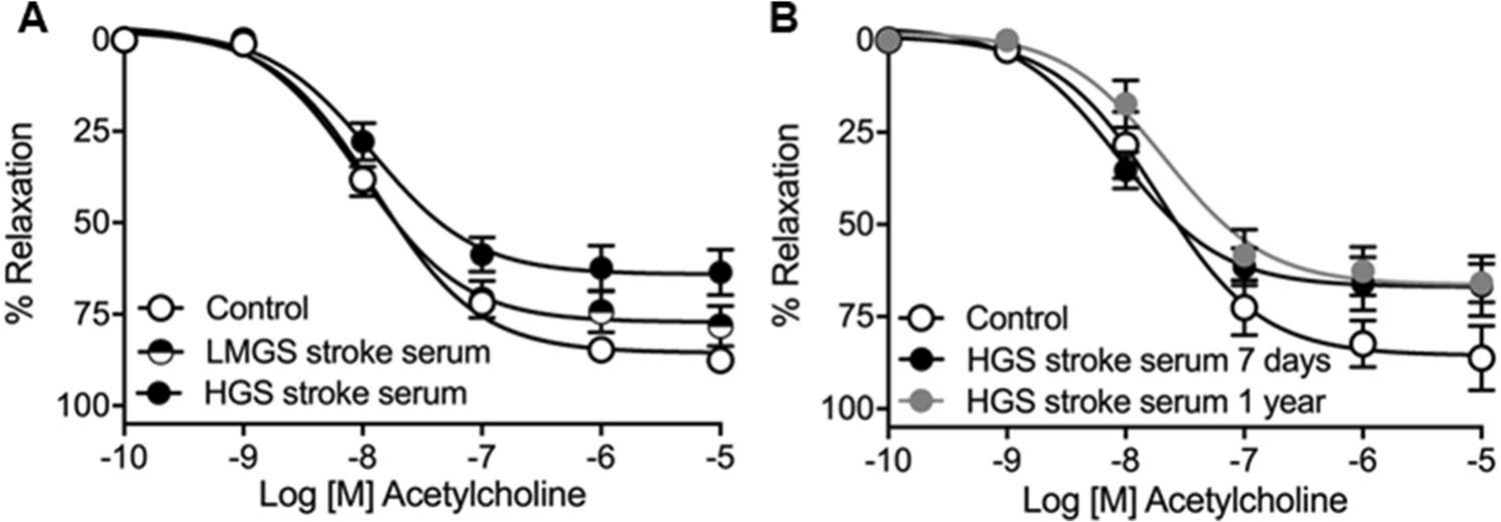
Concentration–response curves to acetylcholine in male OF-1 mice common carotid arteries in the absence (control) or presence (10%) of ischemic stroke serum. Vessels were exposed to serum from ischemic stroke patients (within 7 days poststroke) with low-to-moderate degree (LMGS stroke serum) or high-degree (HGS stroke serum) carotid stenosis (**A**) and with HGS stroke serum from the same patients obtained within 7 days or at 1-year poststroke (**B**). The results are mean ± SEM of (**A**) *n* = 26 (control), 20 (LMGS stroke serum), 10 (HGS stroke serum), and of (**B**) *n* = 6 (HGS stroke serum 7 days) and *n* = 6 (HGS stroke serum 1 year)

Incubation of carotid arteries with either LMGS or HGS stroke serum significantly reduced the maximum vasodilator response to ACh in comparison with vessels (control) not exposed to serum (Fig. 2A; Table 4). Importantly, serum from HGS stroke patients further reduced (*P* < 0.01) the maximum response to ACh compared to LMGS stroke serum (Fig. 2A; Table 4).

**Table 4:** Potency (*pEC*_*50*_) and maximal response (*E*_*max*_) were obtained from concentration–response curves of acetylcholine (ACh) in mice carotid arteries in the absence (control) or presence (10%) of stroke serum from patients with low to moderate-grade (LMGS) or high-grade (HGS) stenosis

	Control (26)	LMGS stroke serum (20)	HGS stroke serum (10)
*pEC*_*50*_	7.89 ± 0.05	8.02 ± 0.10	7.92 ± 0.13
*E*_*max*_	85.54 ± 1.49	77.17 ± 2.59^*^	64.12 ± 2.87^****##^

The results are mean ± SEM, and the number of vessels is shown in parentheses. ^*^P < 0.05; ^****^P < 0.0001 vs. control; ^##^*P* < 0.01 *vs.* LMGS stroke serum by 1-way ANOVA followed by Tukey’s posttest

To elucidate whether the observed deleterious effects of HGS stroke serum (within 7 days from stroke onset) were maintained over time, s obtained 1 year after stroke onset decreased ACh relaxations (i.e., lower *Emax*; *P* < 0.01) to a similar extent than HGS stroke serum obtained within 7-days poststroke (Fig. 2B; Table 5). These results indicate that the capacity of HGS stroke serum to induce mice carotid artery vasodilator dysfunction is maintained until 1 year after the stroke episode.

**Table 5:** Potency (*pEC*_*50*_) and maximal response (*E*_*max*_) were obtained from concentration–response curves of acetylcholine (ACh) in mice carotid arteries in the absence (control) or presence (10%) of stroke serum from high grade stenosis (HGS) patients obtained 7 days or 1 year after the first stroke episode

	Control (6)	HGS stroke serum 7 days (6)	HGS stroke serum 1 year (6)
*pEC*_*50*_	7.72 ± 0.16	8.06 ± 0.15	7.67 ± 0.16
*E*_*max*_	85.52 ± 4.27	66.94 ± 3.2^**^	66.39 ± 3.44^**^

The results are mean ± SEM and the number of vessels is shown in parentheses. ^**^P < 0.01 *vs.* Control by 1-way ANOVA followed by Tukey’s posttest

### HGS Stroke Serum Does Not Alter SNP-Induced Vasodilation

At this stage, we were interested in evaluating the molecular mechanisms involved in mice’s carotid artery ACh vasodilator dysfunction caused by HGS stroke serum (within 7 days from stroke onset). Firstly, we performed concentration–response curves to the NO donor SNP to evaluate smooth muscle cell-dependent relaxations. Arteries incubated with HGS stroke serum exhibited similar levels of relaxation to SNP than control vessels, suggesting that compromised ACh relaxations induced by HGS stroke serum indicate endothelium dysfunction (Supplementary Fig. 2; Supplementary Table 3).

## HGS Stroke Serum-Induced Endothelial Dysfunction Is Not Prevented by NOS Inhibition

**Fig. 3:**
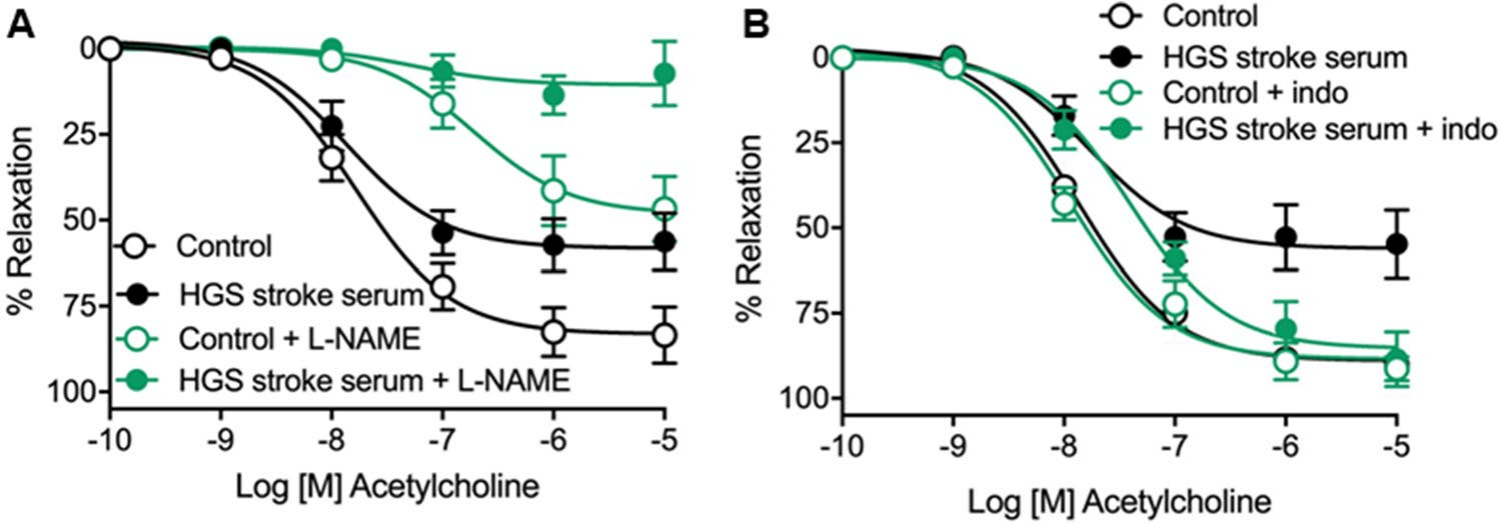
Concentration–response curves to acetylcholine in male OF-1 mice common carotid arteries incubated in the absence (control) or presence (10%) of high-grade stenosis (HGS) stroke serum. Vessels were treated with the nonselective NO synthase inhibitor, L-NAME (300 µM) (**A**), or the nonselective COX inhibitor, indomethacin (indo, 10 µM) (**B**). The results are mean ± SEM of *n* = 6 (**A**) and *n* = 5 (**B**) per group

Nitric oxide is an important mediator of endothelium-dependent relaxation in mice carotid arteries [25]. We used the nonselective NOS inhibitor L-NAME (300 µM) to evaluate whether NO signaling pathway is involved in HGS stroke serum-induced endothelial dysfunction. As expected, NOS inhibition by L-NAME largely decreased carotid artery ACh vasodilation in all vessels (Fig. 3A; Table 6). However, HGS stroke serum-induced impairment of ACh relaxations was still observed in the presence of L-NAME, as in these conditions, *Emax* values were still lower (*P* < 0.0001) than the control group (Fig. 3A; Table 6).

**Table 6:** Potency (*pEC*_*50*_) and maximal response (*E*_*max*_) were obtained from concentration–response curves of acetylcholine (ACh) in mice carotid arteries in the absence (control) or presence (10%) of acute-stroke serum from patients with high-grade (HGS) stenosis and treated with L-NAME (300 µM) or indomethacin (10 µM)

	L-NAME		Indomethacin	
	Control (6)	HGS stroke serum (6)	Control (5)	HGS stroke serum (5)
*pEC*_*50*_	6.72 ± 0.28	7.26 ± 0.91	7.94 ± 0.10	7.41 ± 0.14
*E*_*max*_	48.21 ± 5.71	10.51 ± 3.63^****^	88.42 ± 2.90 (5)	85.35 ± 4.00

The results are mean ± SEM and number of vessels is shown in parentheses. ^****^P < 0.0001 *vs.* control by 1-way ANOVA followed by Tukey’s posttest

## HGS Stroke Serum-Induced Endothelial Dysfunction Is Prevented by COX Inhibition

It is known that altered production of COX-derived prostanoids contributes to the endothelial dysfunction, and a robust body of evidence suggests that COX signaling participates in the pathogenesis of atherosclerotic disease [21]. Thus, we subsequently incubated the vessels with the nonselective COX inhibitor indomethacin (10 µM), which did not affect ACh responses in the control group. Contrariwise, indomethacin prevented the HGS stroke serum-induced endothelial dysfunction (Fig. 3B; Table 6).

**Fig. 4:**
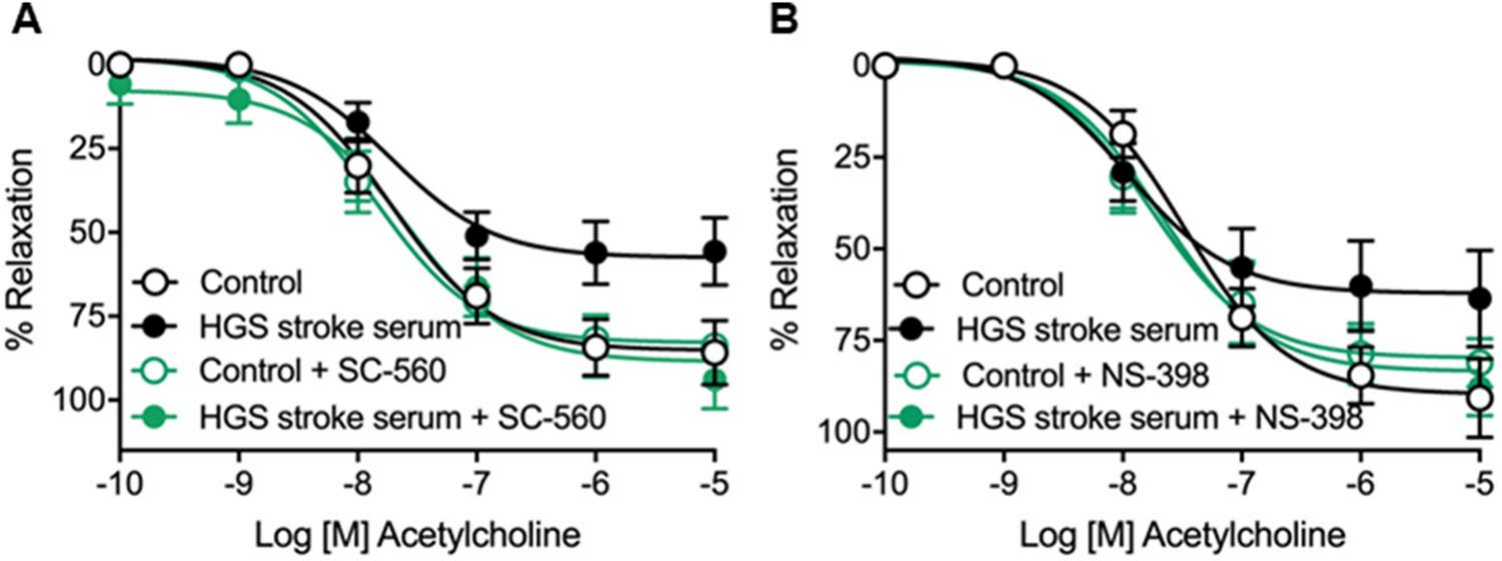
Concentration–response curves to acetylcholine in male OF-1 mice common carotid arteries in the absence (control) or presence (10%) of high-grade stenosis (HGS) stroke serum. Vessels were treated with the selective COX-1 inhibitor, SC-560 (0.3 µM) (**A**), or the selective COX-2 inhibitor, NS-398 (1 µM) (**B**). Data are presented as mean ± SEM of *n* = 5 (**A** and **B**) per group

To elucidate the specific COX isoform involved in endothelial dysfunction, we treated vessels with the selective COX-1 inhibitor, SC-560 (0.3 µM), or the selective COX-2 inhibitor, NS-398 (1 µM). Interestingly, although selective inhibition of either COX-1 (Fig. 4A) or COX-2 (Fig. 4B) did not affect ACh responses in the control group, it prevented HGS stroke serum-induced endothelial dysfunction (Table 7).

**Table 7:** Potency (*pEC*_*50*_) and maximal response (*E*_*max*_) were obtained from concentration–response curves of acetylcholine (ACh) in mice carotid arteries in the absence (control) or presence (10%) of acute-stroke serum from patients with high-grade (HGS) stenosis and treated with SC-560 (0.3 µM) or NS-398 (1 µM)

	SC-560		NS-398	
	Control (5)	HGS stroke serum (5)	Control (5)	HGS stroke serum (5)
*pEC*_*50*_	7.86 ± 0.08	7.58 ± 0.22	7.77 ± 0.18	7.67 ± 0.19
*E*_*max*_	82.88 ± 2.08	88.55 ± 5.63	79.75 ± 4.70 (5)	83.44 ± 5.03

The results are mean ± SEM and number of vessels is shown in parentheses

### Increased IL-1β Levels in HGS Stroke Plasma

**Fig. 5:**
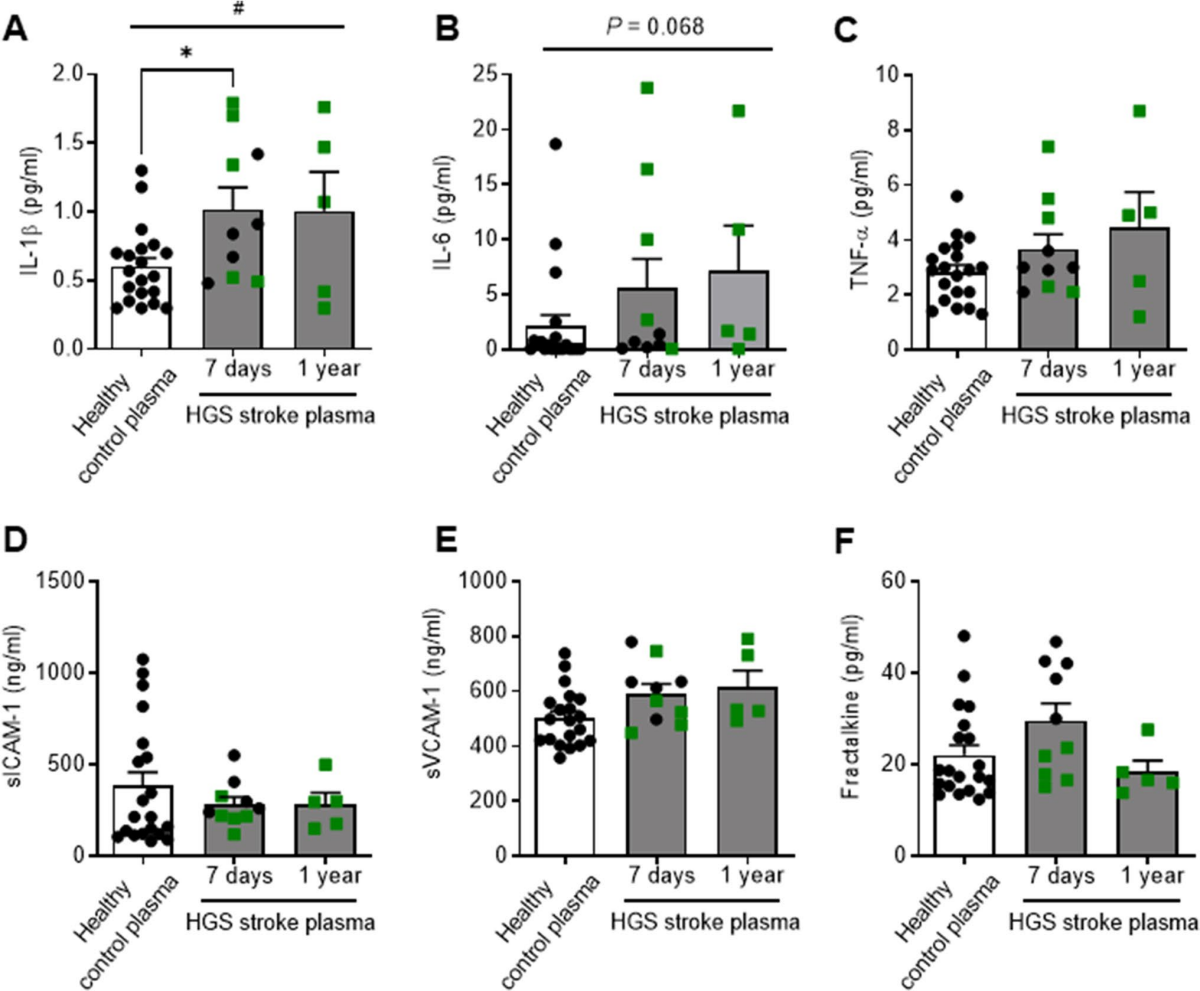
Concentration of circulating inflammatory molecules in healthy control plasma and in plasma from patients with high-grade stenosis (HGS stroke plasma) obtained within 7 days or at 1-year poststroke. The levels of IL-1β (**A**), IL-6 (**B**), TNF-α (**C**), sICAM-1 (**D**), sVCAM-1 (**E**), and fractalkine (**F**) are shown. Data highlighted in green squares indicate samples from the same patients. Data are presented as mean ± SEM of *n* = 20 (healthy control plasma), *n* = 10 (HGS stroke plasma 7 days), and *n* = 5 (HGS stroke plasma 1 year); *P < 0.05 vs. Healthy control plasma by one-way ANOVA with Tukey’s post hoc test; #*P* < 0.05 one-way ANOVA

We reanalyzed plasma IL-1β, IL-6, TNF-α, sICAM-1, sVCAM-1, and FKN levels data obtained in a previous study [17] to test potential differences between HGS stroke patients (within 7 days or 1-year from stroke onset) and healthy controls (Fig. 5). A significant increase in IL-1β levels was observed in HGS stroke patients compared to healthy controls, an effect that was maintained 1-year after stroke (Fig. 5A). In contrast, the levels of the other cytokines and soluble adhesion molecules remained unchanged.

## Discussion

Circulating pro-inflammatory factors released before and after stroke may influence vascular tone in a way that can impair stroke outcomes. In this inflammatory scenario, severe atherosclerotic plaque carotid stenosis may lead to an increased risk of further cerebrovascular events. The present study shows that exposure of carotid arteries from male nonischemic mice with serum from males who have recently had an ischemic stroke induces endothelial dysfunction, an effect that is linked to further adverse clinical events. Notably, we show for the first time that these detrimental effects of serum are more marked in stroke patients with HGS and are linked to increased COX activation in the carotid artery wall. These HGS stroke-induced effects occur in an environment of enhanced circulating levels of IL-1β, which suggests a link between severe carotid narrowing, inflammation, and carotid artery endothelial dysfunction. We propose that locally targeting increased COX activity in the carotid artery wall may be a promising strategy to limit stroke pathophysiology in patients with severe stenosis.

The fact that serum from ischemic stroke patients induces endothelial dysfunction may have clinical significance in patients with adverse vascular events during the follow-up (stroke recurrence or vascular death), whose serum was able to promote more marked carotid artery endothelial dysfunction. One limitation of the present study is that, although tissue baths to measure isometric tension are a widely used method for the “ex vivo” assessment of endothelial function, they do not completely reflect the “in vivo” scenario, where the circulating factors mainly affect the luminal face of the vessel. Nevertheless, the present findings agree with previous literature that indicates endothelial dysfunction has a predictive value for clinical adverse events [29]. In the plaque environment, endothelial dysfunction may result in endothelial cell detachment that may expose the subendothelial matrix, which in conjunction with local neutrophil-induced protease production, can enhance the risk of thrombosis [11]. Notably, when stroke patients were stratified by the degree of carotid stenosis, serum from HGS (≥ 70%) patients was able to promote greater endothelial dysfunction. It is worth noting that 50% of patients with HGS received carotid revascularization and, therefore, the incidence of adverse vascular events may be subject to a certain selection bias. Regarding plasma lipid profile, stroke patients only showed differences in triglycerides, whose increased levels are known to impair endothelial function [30]. However, despite no differences were observed in low-density lipoprotein c levels, an increased proportion of modified low-density lipoproteins leading to inflammation and endothelial dysfunction [10, 31] cannot be discarded and deserve future investigations. Besides, mounting evidence indicates that the increased risk of recurrence in stroke patients with carotid stenosis cannot be explained only by plaque size but also by other crucial factors, such as plaque inflammation and lipid deposition [5, 11, 13], which could contribute in its turn to endothelial dysfunction.

Altered release of circulating vasoactive factors after stroke has been proposed as a possible contributor to impaired poststroke outcomes [14, 32]. Previous works showed that serum from stroke patients induces endothelial dysfunction likely because of pro-oxidative changes in male Wistar Kyoto rat cerebral and mesenteric arteries [19] or leukocyte-derived IL-6 production in the male Sprague–Dawley rat aorta [20]. Similarly, an important contributor to the susceptibility of stroke in atherosclerosis has been linked to the inflammatory response that occurs during the development of the atherosclerotic plaque [12]. Different types of immune cells, namely, macrophages and T lymphocytes, and molecules such as lipoproteins are recruited into the intima and participate in the development of plaque inflammation [13]. For example, oxidized lipoproteins can generate various pro-inflammatory molecules that cannot only influence surrounding cells to increase carotid plaque vulnerability, but also can be released into the circulation to amplify inflammation in a vicious cycle [13]. Thus, inflammation seems to play critical roles in both atherosclerosis and stroke pathophysiology, an effect that may aggravate progression of both diseases in a feed-forward fashion. In the present work, the capacity of HGS stroke serum to induce endothelial dysfunction was maintained 1 year after stroke onset, suggesting that the effect of serum is likely more related to the presence of atherosclerosis and basal chronic inflammation rather than stroke. The results also suggest that despite receiving clinical treatments during poststroke recovery (antiplatelet agents and/or statins), these strategies were not effective to prevent the observed effects mediated by serum. Altogether, the present findings suggest that severe carotid narrowing is linked to greater carotid artery endothelial dysfunction, an effect that can potentially contribute to poststroke pathogenesis.

Plaque macrophages increase the expression of proinflammatory cytokines such as IL-6, IL-1β, and TNF-α, which are the first-wave inflammatory cytokines that impact atherosclerosis substantially [11, 33]. Likewise, increased plasma levels of IL-1β, IL-6, and TNF-α have been reported in ischemic stroke patients [13]. However, contrasting results are available in the literature showing that this triad of cytokines is not always jointly upregulated [14, 34–37]. Discrepancies between studies may depend on different factors including differences in ischemic stroke type, severity of atherosclerosis and stroke, time from stroke onset, poststroke treatments, gender differences, or methods used for cytokine quantification. The present results show that HGS stroke patients have significantly higher circulating levels of IL-1β, whereas IL-6 levels only showed a trend to increase and TNF-α levels were unchanged. Previous studies reported that IL-1β levels are increased both in the plaque and plasma of patients with carotid vulnerable plaques [38], which is associated with higher risk of stroke recurrence [39]. Consistent with the capacity of HGS stroke serum to induce endothelial dysfunction 1 year after stroke onset, IL-1β levels were significantly elevated at this long-term stage, confirming that the effect of serum is likely associated with atherosclerosis rather than stroke.

A recent work that used samples from pooled population of the present observational study (NCT03218527) showed that plasma levels of sICAM-1, sVCAM-1, and FKN were independently associated with plaque inflammation [17]. In particular, sICAM-1 was associated with stroke recurrence regardless of the stenosis degree [17]. Here, we did not observe this expected relationship as sICAM-1, sVCAM-1, and FKN levels were unaltered in HGS stroke patients respect to healthy controls. We speculate that factors such as the lower number of patients, different stenosis grading thresholds (70% vs. 50%), or the exclusion of women in the present study might contribute to the discrepancy. In fact, a major limitation of our study is that studied cohorts are rather small and only men were evaluated. Nonetheless, the present findings provide a starting point for the examination of the impact of severe carotid stenosis on carotid artery endothelial function in both gender larger cohorts.

Interleukin-1β is involved in multiple functions such as cell proliferation, differentiation, and apoptosis and possesses a central role in human atherosclerosis [14]. This cytokine triggers the release of other pro-inflammatory molecules such as IL-6 as well as pro-inflammatory prostanoids [11, 15]. In vessels, exposure to IL-1β exerts pro-oxidative actions leading to endothelial dysfunction [40]. Here, HGS stroke serum-induced endothelial dysfunction was not associated with alterations in the NO signaling pathway, but with COX activation. An imbalance between thromboxane A2 and prostacyclin has a critical role in the development of endothelial dysfunction, and activation of thromboxane A2 receptors has been involved in promoting these detrimental effects [21]. In addition, either pharmacological antagonism or deletion of the thromboxane/prostaglandin receptor limit lesion development in atherosclerosis [41]. On the other hand, cerebral ischemia/reperfusion-induced endothelial dysfunction in rat mesenteric resistance arteries is associated with increased plasma levels of IL-6 and COX activation [42]. We found that either nonselective or selective inhibition of both COX isoforms was sufficient to prevent endothelium dysfunction, which suggests a crosstalk between both COX isoforms in causing this effect. Overall, these findings propose that COX activation may act as a pathological driver of carotid artery endothelial dysfunction in atherothrombotic stroke. Of note, previous studies have shown beneficial effects of systemic anti-inflammatory treatments on stroke outcomes [43]. However, of concern are the clinical setting observations that some anti-inflammatory treatments may increase the susceptibility to fatal infections [15]. Additionally, the IL-1β antagonist canakinumab did not reduce recurrent vascular events in patients with stroke in the CANTOS trial [15]. The present work suggests that instead of blocking systemic inflammation, disrupting the consequences of the inflammatory process directly on the arterial wall, namely, targeting increased COX signaling, might be potentially safer and more effective.

Cyclooxigenase-1 has been classically considered as the isoform primarily responsible for homeostatic prostanoid synthesis, though growing evidence indicates that it can also play important roles in both neurodegenerative [22] and cardiovascular [23] disease. Thus, it is known that IL-1β activates COX-1 to upregulate prostaglandin E2 levels in a transgenic model for conditional and chronic upregulation of this cytokine expression in the hippocampus [44]. Besides, IL-1β-induced increase in prostaglandin H2 by human umbilical vein endothelial cells is suppressed by selective inhibition of COX-2 [45]. In addition, IL-1β stimulation induces COX-2 upregulation in different types of cells, and some studies suggested that this effect could be rapid (1 h) in human vascular smooth muscle cells [46]. In this regard, a rapid endothelium-dependent upregulation of COX-1 and mainly COX-2 leading to thromboxane A2 synthesis was observed after 1 h exposure to IL-1β in rat superior mesenteric arteries [47]. In that study, COX was more rapidly upregulated than iNOS [47], which is consistent with the notion that early production of prostanoids can subsequently stimulate iNOS expression [48].

In conclusion, the present study shows that serum from severe carotid artery stenosis patients with stroke can promote carotid artery endothelial dysfunction linked to increased COX activation. Endothelial dysfunction in an environment of severe carotid stenosis and amplified inflammation may compromise blood supply to the brain and predispose to plaque rupture, leading to a greater risk of further cerebrovascular events. The present findings propose that targeting increased COX signaling locally in the carotid artery wall may have therapeutic benefits for the secondary prevention of atherothrombotic ischemic stroke.

### Supplementary Information

Below is the link to the electronic supplementary material.

Supplementary file1 (PPTX 226 KB)

Supplementary file2 (PPTX 143 KB)

Supplementary file3 (DOCX 24 KB)

Supplementary file4 (DOCX 23 KB)

Supplementary file5 (DOCX 23 KB)

## Data Availability

The data that support the findings of this study are available from the corresponding author upon reasonable request.

## References

[1] GBD 2016 Stroke Collaborators. Global, regional, and national burden of stroke, 1990-2016: a systematic analysis for the Global Burden of Disease Study 2016. Lancet Neurol. 2019;18:439–58 10.1016/S1474-4422(19)30034-1 PMC6494974 30871944

[2] Flach C, Muruet W, Wolfe CDA, Bhalla A, Douiri A (2020). Risk and secondary prevention of stroke recurrence: a population-base cohort study. Stroke.

[3] Lovett JK, Coull AJ, Rothwell PM (2004). Early risk of recurrence by subtype of ischemic stroke in population-based incidence studies. Neurology.

[4] Marulanda-Ledoño E, Chaturvedi S (2016). Stroke due to large vessel atherosclerosis: five new things. Neurol Clin Pract.

[5] Naylor AR, Ricco JB, de Borst GJ, Debus S, de Haro J, Halliday A, Hamilton G, Kakisis J, Kakkos S, Lepidi S (2018). Editor's choice - management of atherosclerotic carotid and vertebral artery disease: 2017 clinical practice guidelines of the European Society for Vascular Surgery (ESVS). Eur J Vasc Endovasc Surg.

[6] Kleindorfer DO, Towfighi A, Chaturvedi S, Cockroft KM, Gutierrez J, Lombardi-Hill D, Kamel H, Kernan WN, Kittner SJ, Leira EC (2021). 2021 Guideline for the prevention of stroke in patients with stroke and transient ischemic attack: a guideline from the American Heart Association/American Stroke Association. Stroke.

[7] Naylor AR (2015). Why is the management of asymptomatic carotid disease so controversial?. Surgeon.

[8] Taylor DW (1991). Beneficial effect of carotid endarterectomy in symptomatic patients with high-grade carotid stenosis. N Engl J Med.

[9] Barnett HJ, Taylor DW, Eliasziw M, Fox AJ, Ferguson GG, Haynes RB (1998). Benefit of carotid endarterectomy in patients with symptomatic moderate or severe stenosis. North American Symptomatic Carotid Endarterectomy Trial Collaborators. N Engl J Med..

[10] Malekmohammad K, Bezsonov EE, Rafieian-Kopaei M (2021). Role of Lipid Accumulation and inflammation in atherosclerosis: focus on molecular and cellular mechanisms. Front Cardiovasc Med.

[11] Hansson GK, Libby P, Tabas I (2015). Inflammation and plaque vulnerability. J Intern Med.

[12] Gimbrone MA, García-Cardeña G (2016). Endothelial cell dysfunction and the pathobiology of atherosclerosis. Circ Res.

[13] Puig N, Jiménez-Xarrié E, Camps-Renom P, Benitez S (2020). Search for reliable circulating biomarkers to predict carotid plaque vulnerability. Int J Mol Sci.

[14] Li X, Lin S, Chen X, Huang W, Li Q, Zhang H, Chen X, Yang S, Jin K, Shao B (2019). The prognostic value of serum cytokines in patients with acute ischemic stroke. Aging Dis.

[15] Libby P (2017). Interleukin-1 beta as a target for atherosclerosis therapy: biological basis of CANTOS and beyond. J Am Coll Cardiol.

[16] Ridker PM, Everett BM, Thuren T, MacFadyen JG, Chang WH, Ballantyne C, Fonseca F, Nicolau J, Koenig W, Anker SD (2017). Antiinflammatory therapy with canakinumab for atherosclerotic disease. N Engl J Med.

[17] Puig N, Camps-Renom P, Camacho M, Aguilera-Simón A, Jiménez-Altayó F, Fernández-León A, Marín R, Martí-Fàbregas J, Sánchez-Quesada JL, Jiménez-Xarrié E, Benitez S (2022). Plasma sICAM-1 as a Biomarker of Carotid Plaque Inflammation in Patients with a Recent Ischemic Stroke. Transl Stroke Res.

[18] Zhang C (2008). The role of inflammatory cytokines in endothelial dysfunction. Basic Res Cardiol.

[19] Canavero I, Sherburne HA, Tremble SM, Clark WM, Cipolla MJ (2016). Effects of acute stroke serum on non-ischemic cerebral and mesenteric vascular function. Transl Stroke Res.

[20] Asano S, O'Connell GC, Lemaster KC, DeVallance ER, Branyan KW, Simpkins JW, Frisbee JC, Barr TL, Chantler PD (2017). Circulating leucocytes perpetuate stroke-induced aortic dysfunction. Exp Physiol.

[21] Mitchell JA, Kirkby NS, Ahmetaj-Shala B, Armstrong PC, Crescente M, Ferreira P, Lopes Pires ME, Vaja R, Warner TD (2021). Cyclooxygenases and the cardiovascular system. Pharmacol Ther.

[22] Ghazanfari N, van Waarde A, Dierckx RAJO, Doorduin J, de Vries EFJ (2021). Is cyclooxygenase-1 involved in neuroinflammation?. J Neurosci Res.

[23] Mitchell JA, Shala F, Pires MEL, Loy RY, Ravendren A, Benson J, Urquhart P, Nicolaou A, Herschman HR, Kirkby NS (2021). Endothelial cyclooxygenase-1 paradoxically drives local vasoconstriction and atherogenesis despite underpinning prostacyclin generation. Sci Adv..

[24] Adams Jr HP, Bendixen BH, Kappelle LJ, Biller J, Love BB, Gordon DL, Marsh 3rd EE. Classification of subtype of acute ischemic stroke. Definitions for use in a multicenter clinical trial. Stroke 1993; 24: 35–41 10.1161/01.str.24.1.35 7678184

[25] Costa TJ, Jiménez-Altayó F, Echem C, Akamine EH, Tostes R, Vila E, Dantas AP, Carvalho MHC (2019). Late onset of estrogen therapy impairs carotid function of senescent females in association with altered prostanoid balance and upregulation of the variant ERα36. Cells.

[26] Chrissobolis S, Didion SP, Kinzenbaw DA, Schrader LI, Dayal S, Lentz SR, Faraci FM (2008). Glutathione peroxidase-1 plays a major role in protecting against angiotensin II-induced vascular dysfunction. Hypertension.

[27] Smith CJ, Zhang Y, Koboldt CM, Muhammad J, Zweifel BS, Shaffer A, Talley JJ, Masferrer JL, Seibert K, Isakson PC (1998). Pharmacological analysis of cyclooxygenase-1 in inflammation. Proc Natl Acad Sci U S A.

[28] Futaki N, Takahashi S, Yokoyama M, Arai I, Higuchi S, Otomo S (1994). NS-398, a new anti-inflammatory agent, selectively inhibits prostaglandin G/H synthase/cyclooxygenase (COX-2) activity in vitro. Prostaglandins.

[29] Esper RJ, Nordaby RA, Vilariño JO, Paragano A, Cacharrón JL, Machado RA (2006). Endothelial dysfunction: a comprehensive appraisal. Cardiovasc Diabetol.

[30] Kajikawa M, Higashi Y (2019). Triglycerides and endothelial function: molecular biology to clinical perspective. Curr Opin Lipidol.

[31] Schnitzler JG, Dallinga-Thie GM, Kroon J (2019). The role of (modified) lipoproteins in vascular function: a duet between monocytes and the endothelium. Curr Med Chem.

[32] Smith CJ, Emsley HC, Gavin CM, Georgiou RF, Vail A, Barberan EM, del Zoppo GJ, Hallenbeck JM, Rothwell NJ, Hopkins SJ (2004). Peak plasma interleukin-6 and other peripheral markers of inflammation in the first week of ischaemic stroke correlates with brain infarct volume, stroke severity and long-term outcome. BMC Neurol.

[33] Winkels H, Ehinger E, Vassallo M, Buscher K, Dinh HQ, Kobiyama K, Hamers AAJ, Cochain C, Vafadarnejad E, Saliba AE (2018). Atlas of the immune cell repertoire in mouse atherosclerosis defined by single-cell RNA-sequencing and mass cytometry. Circ Res.

[34] Fassbender K, Rossol S, Kammer T, Daffertshofer M, Wirth S, Dollman M, Hennerici M (1994). Proinflammatory cytokines in serum of patients with acute cerebral ischemia: kinetics of secretion and relation to the extent of brain damage and outcome of disease. J Neurol Sci.

[35] Tarkowski E, Rosengren L, Blomstrand C, Wikkelsö C, Jensen C, Ekholm S, Tarkowski A (1995). Early intrathecal production of interleukin-6 predicts the size of brain lesion in stroke. Stroke.

[36] Vila N, Filella X, Deulofeu R, Ascaso C, Abellana R, Chamorro A (1999). Cytokine-induced inflammation and long-term stroke functional outcome. J Neurol Sci.

[37] Leira R, Dávalos A, Aneiros Á, Serena J, Pumar JM, Castillo J (2002). Headache as a surrogate marker of the molecular mechanisms implicated in progressing stroke. Cephalalgia.

[38] Shi X, Xie WL, Kong WW, Chen D, Qu P (2015). Expression of the nlrp3 inflammasome in carotid atherosclerosis. J Stroke Cerebrovasc Dis.

[39] Welsh P, Lowe GD, Chalmers J, Campbell DJ, Rumley A, Neal BC, MacMahon SW, Woodward M (2008). Associations of proinflammatory cytokines with the risk of recurrent stroke. Stroke.

[40] Jiménez-Altayó F, Briones AM, Giraldo J, Planas AM, Salaices M, Vila E (2006). Increased superoxide anion production by interleukin-1beta impairs nitric oxide-mediated relaxation in resistance arteries. J Pharmacol Exp Ther.

[41] Kobayashi T, Tahara Y, Matsumoto M, Iguchi M, Sano H, Murayama T, Arai H, Oida H, Yurugi-Kobayashi T, Yamashita JK (2004). Roles of thromboxane A(2) and prostacyclin in the development of aterosclerosis in apoE-deficient mice. J Clin Invest.

[42] Martinez-Revelles S, Jiménez-Altayó F, Caracuel L, Pérez-Asensio FJ, Planas AM, Vila E (2008). Endothelial dysfunction in rat mesenteric resistance artery after transient middle cerebral artery occlusion. J Pharmacol Exp Ther.

[43] Mizuma A, Yenari MA (2017). Anti-Inflammatory targets for the treatment of reperfusion injury in stroke. Front Neurol.

[44] Matousek SB, Hein AM, Shaftel SS, Olschowka JA, Kyrkanides S, O'Banion MK (2010). Cyclooxygenase-1 mediates prostaglandin E(2) elevation and contextual memory impairment in a model of sustained hippocampal interleukin-1beta expression. J Neurochem.

[45] Camacho M, López-Belmonte J, Vila L (1998). Rate of vasoconstrictor prostanoids released by endothelial cells depends on cyclooxygenase-2 expression and prostaglandin I synthase activity. Circ Res.

[46] Beasley D (1999). COX-2 and cytosolic PLA2 mediate IL-1beta-induced cAMP production in human vascular smooth muscle cells. Am J Physiol.

[47] Yuui K, Kudo R, Kasuda S (2022). Arterial thromboxane A2-induced transient contraction after IL-1β exposure. Eur J Inflamm.

[48] Hori M, Kita M, Torihashi S, Miyamoto S, Won KJ, Sato K, Ozaki H, Karaki H (2001). Upregulation of iNOS by COX-2 in muscularis resident macrophage of rat intestine stimulated with LPS. Am J Physiol Gastrointest Liver Physiol.

