# Supplementary material for: Serum from Stroke Patients with High-Grade Carotid Stenosis Promotes Cyclooxygenase-Dependent Endothelial Dysfunction in Non-ischemic Mice Carotid Arteries
Source: Transl Stroke Res. 2022 Dec 19;15(1):140–52. doi: 10.1007/s12975-022-01117-1 (PMC10796474; doi:10.1007/s12975-022-01117-1)
Supplement: Supplementary file 2 — Supplementary file2 (PPTX 143 KB) [file 12975_2022_1117_MOESM2_ESM.pptx]

## Slide 1
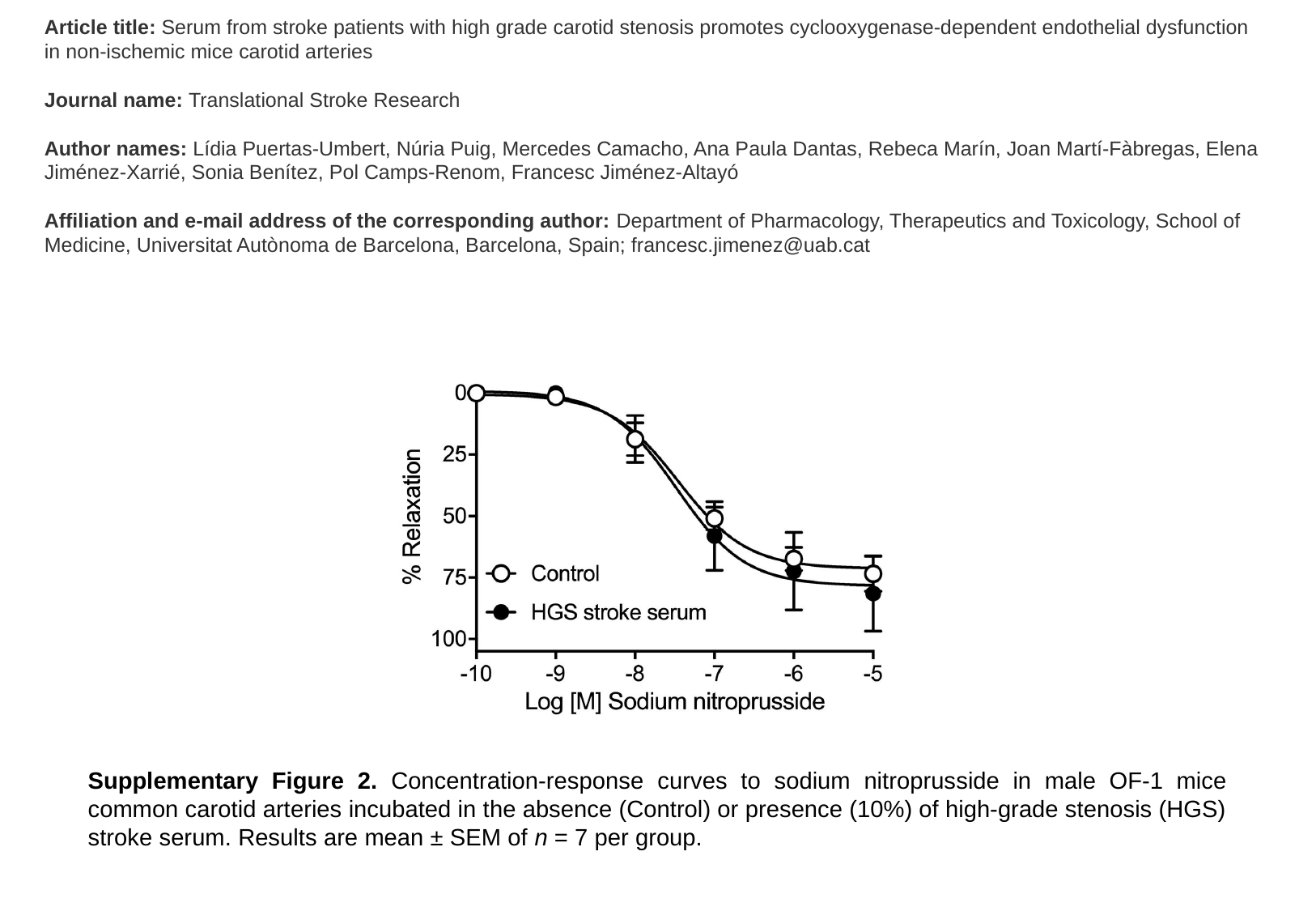

Article title: Serum from stroke patients with high grade carotid stenosis promotes cyclooxygenase-dependent endothelial dysfunction in non-ischemic mice carotid arteries
Journal name: Translational Stroke Research
Author names: Lídia Puertas-Umbert, Núria Puig, Mercedes Camacho, Ana Paula Dantas, Rebeca Marín, Joan Martí-Fàbregas, Elena Jiménez-Xarrié, Sonia Benítez, Pol Camps-Renom, Francesc Jiménez-Altayó
Affiliation and e-mail address of the corresponding author: Department of Pharmacology, Therapeutics and Toxicology, School of Medicine, Universitat Autònoma de Barcelona, Barcelona, Spain; francesc.jimenez@uab.cat
Supplementary Figure 2. Concentration-response curves to sodium nitroprusside in male OF-1 mice common carotid arteries incubated in the absence (Control) or presence (10%) of high-grade stenosis (HGS) stroke serum. Results are mean ± SEM of n = 7 per group.
