# Supplementary material for: Serum from Stroke Patients with High-Grade Carotid Stenosis Promotes Cyclooxygenase-Dependent Endothelial Dysfunction in Non-ischemic Mice Carotid Arteries
Source: Transl Stroke Res. 2022 Dec 19;15(1):140–52. doi: 10.1007/s12975-022-01117-1 (PMC10796474; doi:10.1007/s12975-022-01117-1)
Supplement: Supplementary file 3 — Supplementary file3 (DOCX 24 KB) [file 12975_2022_1117_MOESM3_ESM.docx]

**Article title:** Serum from stroke patients with high grade carotid stenosis promotes cyclooxygenase-dependent endothelial dysfunction in non-ischemic mice carotid arteries

**Journal name:** Translational Stroke Research

**Author names:** Lídia Puertas-Umbert, Núria Puig, Mercedes Camacho, Ana Paula Dantas, Rebeca Marín, Joan Martí-Fàbregas, Elena Jiménez-Xarrié, Sonia Benítez, Pol Camps-Renom, Francesc Jiménez-Altayó

**Affiliation and e-mail address of the corresponding author:** Department of Pharmacology, Therapeutics and Toxicology, School of Medicine, Universitat Autònoma de Barcelona, Barcelona, Spain; francesc.jimenez@uab.cat

| **Supplementary Table 1.**  Potency (pEC_50_) and maximal response (E_max_) were obtained from concentration-response curves of acetylcholine (ACh) in mice carotid arteries in the absence (No serum) or presence (1%, 3%) of stroke serum. | | | | |
| --- | --- | --- | --- | --- |
|  | **No serum (17)** | **1% serum (17)** | **No serum (22)** | **3% serum (22)** |
|  |  |  |  |  |
| **pEC_50_** | 7.95 ± 0.06 | 8.02 ± 0.11 | 7.97 ± 0.05 | 7.86 ± 0.11 |
|  |  |  |  |  |
| **E_max_** | 83.17 ± 2.03 | 84.64 ± 3.87 | 84.79 ± 1.35 | 79.81 ± 2.79 |
| Results are mean ± SEM and number of vessels is shown in parentheses. | | | | |
