# Supplementary material for: Serum from Stroke Patients with High-Grade Carotid Stenosis Promotes Cyclooxygenase-Dependent Endothelial Dysfunction in Non-ischemic Mice Carotid Arteries
Source: Transl Stroke Res. 2022 Dec 19;15(1):140–52. doi: 10.1007/s12975-022-01117-1 (PMC10796474; doi:10.1007/s12975-022-01117-1)
Supplement: Supplementary file 4 — Supplementary file4 (DOCX 23 KB) [file 12975_2022_1117_MOESM4_ESM.docx]

**Article title:** Serum from stroke patients with high grade carotid stenosis promotes cyclooxygenase-dependent endothelial dysfunction in non-ischemic mice carotid arteries

**Journal name:** Translational Stroke Research

**Author names:** Lídia Puertas-Umbert, Núria Puig, Mercedes Camacho, Ana Paula Dantas, Rebeca Marín, Joan Martí-Fàbregas, Elena Jiménez-Xarrié, Sonia Benítez, Pol Camps-Renom, Francesc Jiménez-Altayó

**Affiliation and e-mail address of the corresponding author:** Department of Pharmacology, Therapeutics and Toxicology, School of Medicine, Universitat Autònoma de Barcelona, Barcelona, Spain; francesc.jimenez@uab.cat

| **Supplementary Table 2.** Potency (pEC_50_) and maximal response (E_max_) were obtained from concentration-response curves of acetylcholine (ACh) in mice carotid arteries in the absence (No serum) or presence (10%) of healthy control serum. | | |
| --- | --- | --- |
|  | **No serum (11)** | **Healthy control serum (20)** |
|  |  |  |
| **pEC_50_** | 7.89 ± 0.11 | 7.88 ± 0.11 |
|  |  |  |
| **E_max_** | 73.74 ± 2.73 | 73.24 ± 2.47 |
| Results are mean ± SEM and number of vessels is shown in parentheses. | | |
