# Supplementary material for: Serum from Stroke Patients with High-Grade Carotid Stenosis Promotes Cyclooxygenase-Dependent Endothelial Dysfunction in Non-ischemic Mice Carotid Arteries
Source: Transl Stroke Res. 2022 Dec 19;15(1):140–52. doi: 10.1007/s12975-022-01117-1 (PMC10796474; doi:10.1007/s12975-022-01117-1)
Supplement: Supplementary file 5 — Supplementary file5 (DOCX 23 KB) [file 12975_2022_1117_MOESM5_ESM.docx]

**Article title:** Serum from stroke patients with high grade carotid stenosis promotes cyclooxygenase-dependent endothelial dysfunction in non-ischemic mice carotid arteries

**Journal name:** Translational Stroke Research

**Author names:** Lídia Puertas-Umbert, Núria Puig, Mercedes Camacho, Ana Paula Dantas, Rebeca Marín, Joan Martí-Fàbregas, Elena Jiménez-Xarrié, Sonia Benítez, Pol Camps-Renom, Francesc Jiménez-Altayó

**Affiliation and e-mail address of the corresponding author:** Department of Pharmacology, Therapeutics and Toxicology, School of Medicine, Universitat Autònoma de Barcelona, Barcelona, Spain; francesc.jimenez@uab.cat

| **Supplementary Table 3.** Potency (pEC_50_) and maximal response (E_max_) were obtained from concentration-response curves of sodium nitroprusside in mice carotid arteries in the absence (Control) or presence (10%) of stroke serum from patients with high-grade (HGS) stenosis. | | |
| --- | --- | --- |
|  | **Control (7)** | **HGS stroke serum (7)** |
|  |  |  |
| **pEC_50_** | 7.46 ± 0.15 | 7.48 ± 0.31 |
|  |  |  |
| **E_max_** | 71.34 ± 3.45 | 78.34 ± 8.01 |
| Results are mean ± SEM and number of vessels is shown in parentheses. | | |
